# Modulation of the immune response by *Fonsecaea pedrosoi* morphotypes in the course of experimental chromoblastomycosis and their role on inflammatory response chronicity

**DOI:** 10.1371/journal.pntd.0005461

**Published:** 2017-03-29

**Authors:** Isaque Medeiros Siqueira, Raffael Júnio Araújo de Castro, Luiza Chaves de Miranda Leonhardt, Márcio Sousa Jerônimo, Aluízio Carlos Soares, Tainá Raiol, Christiane Nishibe, Nalvo Almeida, Aldo Henrique Tavares, Christian Hoffmann, Anamelia Lorenzetti Bocca

**Affiliations:** 1 Molecular Pathology Post-Graduate Program, School of Medicine; University of Brasília, Brasília, Brazil; 2 Department of Cell Biology, Institute of Biological Sciences; University of Brasília, Brasília, Brazil; 3 University Hospital, University of Brasília, Brasília, Brazil; 4 Institute Leônidas and Maria Deane, Oswaldo Cruz Foundation, Manaus, Brazil; 5 School of Computing Sciences, Federal University of Mato Grosso do Sul, Campo Grande, Brazil; 6 Department of Food Sciences and Experimental Nutrition, School of Pharmaceutical Sciences, University of São Paulo, São Paulo, Brazil; University of Tennessee, UNITED STATES

## Abstract

A common theme across multiple fungal pathogens is their ability to impair the establishment of a protective immune response. Although early inflammation is beneficial in containing the infection, an uncontrolled inflammatory response is detrimental and may eventually oppose disease eradication. Chromoblastomycosis (CBM), a cutaneous and subcutaneous mycosis, caused by dematiaceous fungi, is capable of inducing a chronic inflammatory response. Muriform cells, the parasitic form of *Fonsecaea pedrosoi*, are highly prevalent in infected tissues, especially in long-standing lesions. In this study we show that hyphae and muriform cells are able to establish a murine CBM with skin lesions and histopathological aspects similar to that found in humans, with muriform cells being the most persistent fungal form, whereas mice infected with conidia do not reach the chronic phase of the disease. Moreover, in injured tissue the presence of hyphae and especially muriform cells, but not conidia, is correlated with intense production of pro-inflammatory cytokines *in vivo*. High-throughput RNA sequencing analysis (RNA-Seq) performed at early time points showed a strong up-regulation of genes related to fungal recognition, cell migration, inflammation, apoptosis and phagocytosis in macrophages exposed *in vitro* to muriform cells, but not conidia. We also demonstrate that only muriform cells required FcγR and Dectin-1 recognition to be internalized *in vitro*, and this is the main fungal form responsible for the intense inflammatory pattern observed in CBM, clarifying the chronic inflammatory reaction observed in most patients. Furthermore, our findings reveal two different fungal-host interaction strategies according to fungal morphotype, highlighting fungal dimorphism as an important key in understanding the bipolar nature of inflammatory response in fungal infections.

## Author summary

Pathogenic fungi often present two distinct forms, which are correlated to the host’s inflammatory response and eventual disease outcome. Chromoblastomycosis is a fungal disease occurring especially in tropical areas, and it is most often caused by the dimorphic fungus *Fonsecaea pedrosoi*. Although it is not a notifiable disease, it is estimated that this disease affects several million people worldwide. Treatment includes long-term antifungal chemotherapy and it is often combined with physical and surgical treatment, even so the relapse ratio is high. We were able to demonstrate the existence of two distinct fungal-host interactions. Muriform cells were responsible for inflammatory response and conidia were related to fungus persistence in the tissue, highlighting fungal dimorphism as an important element in understanding the bipolar nature of the inflammatory response in some fungal infections. These data open avenues to rethinking a better treatment schedule that takes into consideration the host’s immune response to fungal infections.

## Introduction

A common theme observed across multiple fungal pathogens is their ability to impair the establishment of a protective immune response. Although early inflammation is beneficial in containing the infection, an uncontrolled inflammatory response is detrimental and may eventually oppose disease eradication [1,2]. In some clinical settings, disease chronicity may be the result of an exaggerated inflammatory response that probably compromises the host’s ability to cope with infective fungi, as opposed to an ‘intrinsic’ susceptibility to infection [2]. For instance, the host’s inability to control infections by *Aspergillus fumigatus*, *Candida albicans* and *Paracoccidioides brasiliensis* has been linked to a failure in controlling inflammation relating to specific fungal components [1,3,4].

Chromoblastomycosis (CBM) is a cutaneous and subcutaneous mycosis, caused by dematiaceous fungi, which is capable of inducing a chronic inflammatory response, making it a suitable model to study chronic inflammation caused by dimorphic fungal infection. Despite occurring worldwide, it has a high prevalence in humid areas of tropical and subtropical climate [5,6]. *Fonsecaea pedrosoi* is the predominant causative agent of CBM, being found as a saprophyte in soil and plant tissues. Thorns and wood splinters are thought to promote traumatic inoculation of fungal propagules, consisting of fungal conidia and hyphal fragments into host skin, more frequently into lower limbs [6–8]. The incubation period can take several years post-infection, after which time patients slowly develop polymorphic skin lesions including nodules, verrucas and plaques, scar tissues and tumors [8]. CBM patients usually present low disease resolution and high relapse rates after treatment, which includes long-term antifungal chemotherapy and is often combined with physical and surgical treatment [9].

CBM histological examination reveals pseudo-epitheliomatous hyperplasia and highly organized suppurative granulomatous inflammation accompanied by necrosis. It presents dense inflammatory infiltrates, enclosed within a thick fibrous edge, which are rich in granulocytes, especially activated neutrophils [10,11]. Macrophages are regularly observed within the lesions, in different degrees of maturation and activation, and also form multinucleated giant cells, sometimes containing fungal parasitic forms within [10].

Different fungal morphotypes have been associated with different immune response patterns, and the successful establishment of fungal infection in mammalian hosts usually requires the pathogen’s ability to switch between different forms [12]. Furthermore, the ability to exist in different forms and to reversibly switch from one to the other during infection is an important virulence factor, which allows fungal survival and persistence in the host [1,2]. CBM etiologic agents exhibit morphological and biochemical composition changes from saprophytic (hyphal and conidia cells) to parasitic forms denominated muriform cells (also known as sclerotic cells or medlar bodies). These cells are credited as a key contributor to *F*. *pedrosoi* virulence and they are highly prevalent in infected tissues, especially in long-standing lesions [8,13–16]. Muriform cells (MCs) main features include meristematic growth of swollen, thick-walled cells with increased melanin deposition that provides resistance to fungal elimination by phagocytosis [13,17].

In mice infected with fugal conidia, recognition of *F*. *pedrosoi* by cells of the innate immune system occurs mainly through the engagement of the C-type lectin receptors (CLRs) Dectin-1 and Dectin-2 [18]. *F*. *pedrosoi* conidia (FC) are unable to promote the release of pro-inflammatory cytokines, such as TNF-α, which is only re-instated after Toll like receptor (TLR) co-stimulation [19]. Studies of *F*. *pedrosoi*, mostly making use of its conidial form, have thus far been unable to completely elucidate the pathogenesis of CBM, leaving a gap in the understanding of the disease progression and the markedly chronic inflammatory response observed in CBM [9,20–22].

In this study we show that *F*. *pedrosoi* hyphae (FH) and muriform cells are able to establish a murine CBM displaying skin lesions and histopathological features similar to that found in humans, with muriform cells being the most persistent fungal morphotype, whereas mice infected with *F*. *pedrosoi* conidia do not reach the chronic phase of the disease. Furthermore, the presence of hyphae and especially muriform cells, but not conidia, is correlated with intense production of pro-inflammatory cytokines *in vivo*. High-throughput RNA sequencing analysis showed distinct patterns of gene expression dependent on the fungal form interaction used: macrophages exposed to muriform cells, but not conidia, exhibited a strong up-regulation of genes related to fungal recognition, cell migration, inflammation and apoptosis. Altogether, our findings reveal two different fungal-host interaction strategies according to fungal form, highlighting fungal dimorphism as an important key in understanding the bipolar nature of the inflammatory response in fungal infections.

## Materials and methods

### Fungal strain and infection

*F*. *pedrosoi* (ATCC 46428) was cultivated in Sabouraud Dextrose Agar medium (SDA, Himedia) supplemented with 100 mg.l^-1^ chloramphenicol at 37°C, as described previously [23]. Fungal virulence and strain adaptation to an animal host was acquired by sequentially inoculating *F*. *pedrosoi* propagules three times into experimental animal footpads at 2x10^7^ cells per ml (50 μl per foot), followed by strain recovery 15 days later in SDA medium.

**Fig 1:**
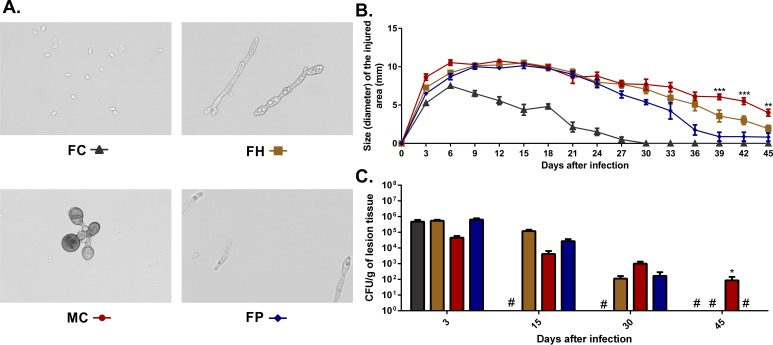
Progression of murine chromoblastomycosis induced by different *F*. *pedrosoi* fungal forms. BALB/c mice were infected in the footpad with 1x10^6^ conidia (FC), hyphae (FH), muriform cells (MC) or a combination of hyphal fragments and conidia (FP) in the ratio of 3:1 (A). Morphometric (B) and CFU data (C) showed a fast clearance of inoculated conidia, while infection with MCs was reflected in the persistence of the fungus in the tissue up to 45 days after infection. 400x magnification (A). *P<0.05 and ***P<0.001 compared to FH group.

Purified conidia and hyphae were obtained by growing virulent *F*. *pedrosoi* propagules in potato dextrose medium in a rotary shaker (120 rpm) at 37°C for 7–14 days. At that time, culture suspensions containing conidia and hyphal fragments were first filtered in sterile fiberglass to remove large hyphae clumps. The filtrate was subjected to successive filtrations on 70 μm and 40 μm cell strainers (BD). Retained hyphae in the 40 μm cell strainer (measuring 40 to 70 μm) were re-suspended in phosphate buffered saline (PBS) and centrifuged twice at 1000 g, providing more than 98% of purified hyphae (Fig 1A, FH). Filtrate containing conidia and small hyphal fragments from the 40 μm cell strainer was further filtered using a 14 μm filter paper (J. Prolab, Brazil), and centrifuged twice at 3000 g, yielding a cell suspension containing at least 98% purified conidia (Fig 1A, FC). Fungal propagules (Fig 1A, FP) were obtained by mixing the purified hyphae and conidia at a 3:1 rate. Muriform cells were obtained from virulent *F*. *pedrosoi* propagule culture in Butterfield’s chemically defined medium (BF) supplemented with propranolol at 37°C at pH 2.7 under 120 rpm, as previously described [24]. The suspension containing *F*. *pedrosoi* muriform cells was then filtered through a 40 μm cell strainer, yielding more than 90% of purified muriform cells (Fig 1A, MC). Live and purified fungal cells were finally counted in a hemocytometer using cotton blue dye and then inoculated into experimental animal footpad at 2x10^7^ conidia, hyphal fragments or muriform cells per ml (50 μl per foot).

### Animals and experimental design

BALB/c mice (male, 6–8 weeks old) were purchased from the University of São Paulo (USP, SP, Brazil) and maintained under standard laboratory conditions. In addition to the healthy group, animals were divided into four experimental groups: **i.** Mice infected with *F*. *pedrosoi* conidia (FC); **ii.** Mice infected with *F*. *pedrosoi* hyphae (FH); **iii.** Mice infected with *F*. *pedrosoi* muriform cell (MC); **iv.** Mice infected with *F*. *pedrosoi* propagules (FP). Eight animals per group were euthanized in CO_2_ chamber, 3, 15, 30 and 45 days after infections. Injured tissue was measured with a caliper and collected for downstream analysis. Zymosan-induced inflammation was performed as described elsewhere [25,26]. After 15 days of infection with FP, 16 animals were treated every three days in the footpad with 20μl of a suspension containing 5 mg/ml of zymosan (Sigma-Aldrich) or PBS. Cell migration to peritoneal cavity was assessed through peritoneal inoculation of 10^6^ cells of each fungal form. After 4 and 72 hours of inoculation, leukocytes were harvested from 8 animals per group and quantified with the aid of Automated Cell Counter.

### Ethics statement

All experimental procedures involving animals were approved by the Ethics Committee for Scientific Studies of the University of Brasilia, and conducted in accordance with the Brazilian Council for the Control of Animal Experiments (CONCEA) guidelines on the use and care of laboratory animals (UnBDoc n^o^. 135976/2014).

### Histopathology, differential fungal quantification and fungal burden

Small fragments of infected tissue were fixed dehydrated and embedded in paraffin to evaluate lesion progression. Serial sections were made and stained with hematoxylin and eosin (HE) or Masson’s trichrome stain. Differential fungal quantification was performed using three histological slides from the same tissue fragment. *F*. *pedrosoi* cells were counted on twenty fields chosen at random in these slides with the aid of a counting reticle. Cell counts were expressed as cells per mm^2^ of injured tissue. Other samples from each experimental animal were also homogenized in PBS (pH 7.2) and then plated onto SDA medium, supplemented with 100 mg.l^-1^ chloramphenicol and cultivated at 37°C for seven days. Fungal burden was then measured by quantitative counts of colony-forming units (CFU) of *F*. *pedrosoi*. Results were expressed as number of CFU ± standard deviation (SD) per gram of tissue.

### Peritoneal macrophages (PMs), bone marrow-derived macrophages (BMDMs) and dendritic cells (BMDCs)

Peritoneal macrophages (PMs) were obtained as described previously [27]. The cell suspension acquired was re-suspended in RPMI medium (with 2% Fetal Bovine Serum (Gibco–Thermo Fisher Scientific)) and 80 μg/ml of gentamicin. Mouse bone marrow-derived macrophages (BMDMs) and dendritic cells (BMDCs) were generated using GM-CSF, as described previously [28], and re-suspended in RPMI medium (with 10% FBS) for subsequent use. 5x10^5^ cells were plated in a 24-well plate and incubated at 37°C, in 5% CO_2_, for 24 hours to allow macrophage adhesion and used in the subsequent fungal-phagocyte co-cultures.

### Fungal–phagocyte co-cultures

Phagocytes were plated and infected with *F*. *pedrosoi* conidia or muriform cells at MOI 1 and co-cultured for 6, 12, 24 and 48h. A positive control culture was prepared using 1 μg/ml LPS (*Escherichia coli* serotype 0111:B, Sigma-Aldrich) and 100 U/ml interferon-gamma (IFN-γ, Sigma-Aldrich). For IL-1β induction, 500 ng/ml LPS and 5 mM ATP (InvivoGen) were used, individually or associated with fungal cells. Cell culture supernatants were collected and subjected to cytokines, chemokine and nitric oxide quantification. Phagocytosis index assessment was made as previously described [29] with or without Fcblock (BD biosciences) or laminarin (InvivoGen). Cells were rinsed to remove non-phagocytosed fungal cells, fixed with absolute methanol and stained with 20% buffered Giemsa solution. The number of attached and/or ingested fungal cells per 200 macrophages was evaluated microscopically in triplicate preparations.

### Measurement of nitric oxide (NO) concentration and cytokine production assay

NO_2_ concentration in culture supernatants was used as an indicator of NO generation and it was measured using Griess reagent as described previously [30].

Cytokine production was measured from homogenized tissue obtained from infected animals as well as cell culture supernatants by Elisa, following manufactory instructions: interleukin-1β (IL-1β), interleukin-6 (IL-6), tumor necrosis factor (TNF), monocyte chemoattractant protein-1 (MCP-1/Ccl2) (eBioscience) and Interleukin-12 (IL-12) (BD Biosciences). The absolute cytokine level present in the samples was calculated based on a standard curve provided by the commercial kit.

### High-throughput RNA sequencing

Peritoneal macrophages (PMs) were infected for 6h with *F*. *pedrosoi* conidia or muriform cells. Washed macrophages were then lysed, and total RNA was extracted with the RNeasy kit (Qiagen) according to the manufacturer's instructions. Extracted RNA was quantified in a fluorometer (Qubit) and submitted to Bioanalyzer 2100 (Agilent) to determine its integrity. Paired-end cDNA reads (100bp) were generated using the HiSeq 2000 Sequencing system (Illumina) located at the Scripps DNA Sequencing Facility (California, USA) according to the manufacturer’s standard protocol. Quality check of the paired-end reads was performed using FASTQC [31], and clipping and trimming was done using CUTADAPT [32] and PRINSEQ [33] software, respectively. The filtered reads were aligned to the mouse genome downloaded from the Ensembl database [34] using open source TopHat 2.0.9 [35]. The aligned files were ordered and indexed using Samtools [36] followed by read count using HTSeq-count [33]. Statistical analysis was done using the R environment for statistical computing [37]. Gene model quantifications were performed using the Bioconductor package EdgeR [38]. Genes were considered as differentially expressed when FDR and corrected p-values were < 0.05 and Fold-change > 1.4. Heatmaps of differentially expressed genes were generated using the package gplots. Genes considered differentially expressed were annotated with their Biological Process gene ontology using the package org.Mm.eg.db [39], placed within pathways using the package pathview [40]. Genes detected to be significantly modulated by the EdgeR analysis were used as input on a Biological Process Gene Ontology enrichment analysis using the R package topGO [41]. Only nodes containing at least 10 genes were considered in the analysis and the classic algorithm was used with the Fisher’s exact test. The p-values obtained were FDR adjusted.

### Accession numbers

All sequencing data are deposited at NCBI's GEO database (GSE 84257).

## Results

### Different *F*. *pedrosoi* fungal forms trigger distinct infection patterns

We infected Balb/c mice in the footpad with 10^6^ cells of each of the fungal morphotypes (conidia, hyphae and muriform cells) (Fig 1A) to determine how different *F*. *pedrosoi* fungal forms trigger CBM establishment and development. Footpad infection with *F*. *pedrosoi* propagules is already known to establish experimental CBM with skin lesions and histopathological features similar to that found in humans [18,23,42,43] and it is used here as a positive control.

Ulcerative lesions similar to those found in humans arose after 15 days in all infected animals, except for those infected with conidia which presented local edema in the first 6 days of infection followed by reduction in the injured area size (Fig 1B). Histopathological analysis of skin lesions of animals infected with hyphae and muriform cells showed ulceration of exudative areas, with the presence of necrotic material and fungal cells, as well as a multifocal lymphocytic infiltrate outlining a granulomatous aspect which is similar to that observed in humans with the disease (S1A Fig). After 30 days of infection, progressive healing in the injured area was evident in all groups. However, after 45 days only animals infected with muriform cells still showed significant edema (Fig 1B).

Fungal recovery from infected footpads revealed a gradual fungal elimination from the infected site. The CFU number was reduced over time in all animals, reaching undetectable levels after 45 days of infection, except for those infected with muriform cells, which still showed fungal recovery at that time (Fig 1C). When taken together with the morphometric and histopathological analysis, these data indicate that different *F*. *pedrosoi* fungal forms trigger distinct infection patterns so that conidia alone was not able to develop CBM characteristic skin lesions in contrast to muriform cells infection which develops a longer-lasting experimental CBM when compared to other *F*. *pedrosoi* morphotypes.

### Presence of muriform cells and hyphae in injured tissue, but not conidia, is correlated with intense proinflammatory cytokines production and cell migration during CBM establishment

**Fig 2:**
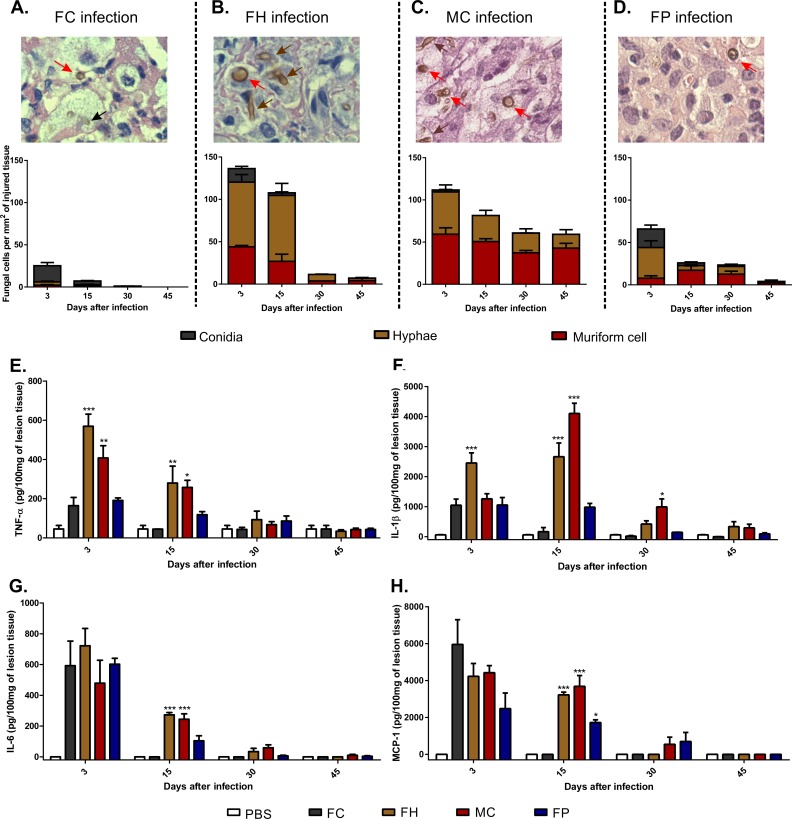
Quantification of *F*. *pedrosoi* fungal cells in the tissue and production of pro-inflammatory cytokines in the course of murine CBM. Fungal cells were counted on twenty fields chosen at random in histopathological slides with the aid of a counting reticle (A-D). In all groups muriform cells (red arrows) could be identified after 15 days after infection. At the same time, hyphal fragments (brown arrow) were also present in animals infected either with hyphae (FH) (B) or muriform cells (MC) (C). Few conidia (black arrow) are observed after 15 days of infection in animals infected with *F*. *pedrosoi* conidia (FC) (A). Cell counts were expressed as cells per mm^2^ of injured tissue. 1000x magnification (A-D). After 15 days of infection, high levels of TNF-α (E), IL-1β (F), IL-6 (G) and MCP-1 (H) were observed in groups with higher numbers of hyphae and muriform cells in the tissue. Cytokine production was measured by ELISA from homogenized footpad tissue. *P<0.05, **P<0.01 and ***P<0.001 compared to FC group.

Quantification of fungal morphotypes in the tissue lesions showed that both conidia and hyphae were able to turn into muriform cells (Fig 2A, 2B and 2D, red arrows). Moreover, it was possible to observe muriform cell germination generating hyphae in the tissue (Fig 2C, brown arrow). Conidia infection induced significantly lower levels of pro-inflammatory cytokines when compared to infections using hyphae and muriform cells, even though some conidia turned into muriform cells (Fig 2A, red arrow). Indeed, hyphae and muriform cells infection induced high levels of TNF-α, IL-1β and IL-6 during the disease establishment stage (Fig 2E–2G). A reduction in proinflammatory cytokines was observed only after 30 days of infection, considered the resolution stage for murine CBM (Fig 2E–2H), and only muriform cells infected animals showed significantly higher levels of IL-1β at this time point (Fig 2F). High levels of MCP-1 were also observed after 15 days of infection in animals inoculated with hyphae or muriform cells (Fig 2H), suggesting that those fungal cells are able to induce strong cell migration in the tissue. Taken together, these results demonstrate that the presence of muriform cells and hyphae in injured tissue, but not conidia, is correlated with an intense production of proinflammatory cytokines and cell migration during establishment of the experimental CBM.

### Muriform cells promote large upregulation of inflammatory response genes

**Fig 3:**
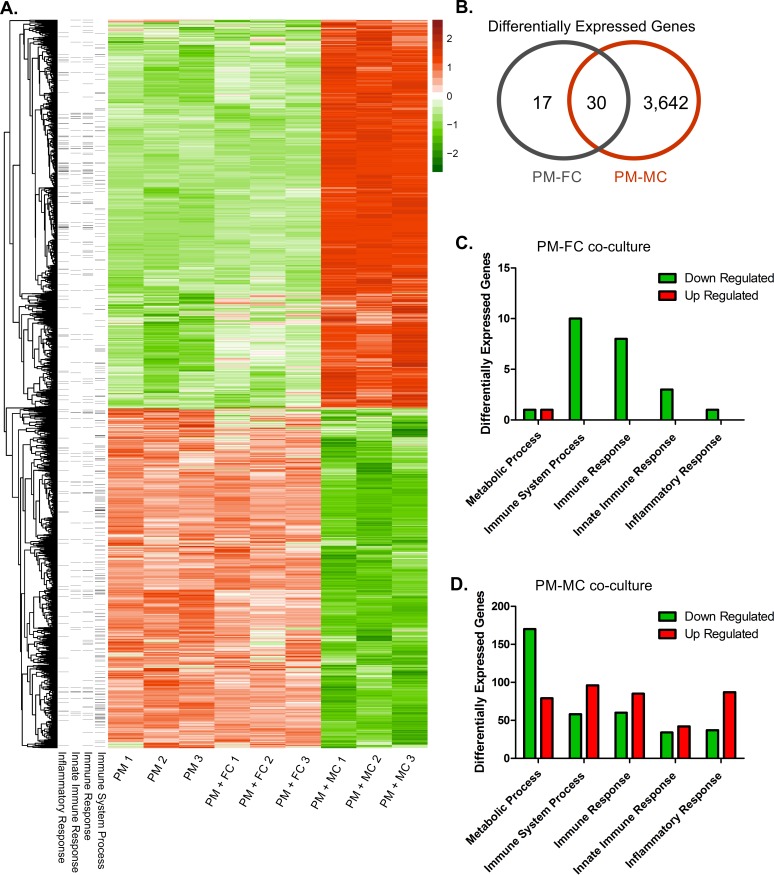
Differential expression of genes in peritoneal macrophages after co-cultivation with conidia or muriform cells. Global heatmap displaying all differentially expressed genes (considering each replicate) showed profound changes in peritoneal macrophages (PM) gene expression profile when stimulated with muriform cells (MC), while conidia (FC) and macrophage co-culture showed similar patterns to that found in unstimulated cells (A). Heatmap was built based on z-score ranking considering all genes considered as differentially expressed. Venn diagram (B) shows high number of differentially expressed genes (3672 genes) when macrophages were co-cultured with muriform cells, while only 47 genes were differentially expressed in conidia and macrophage co-culture. GO enrichment analysis in FC-PM interaction showed few differentially expressed genes correlated to immune response (C). On the other hand, MC-PM interaction promoted the up-regulation of a large number of genes related to the immune system, especially those concerning the inflammatory response (D).

As different *F*. *pedrosoi* fungal morphotypes were able to activate distinct inflammatory response patterns *in vivo*, we further evaluated the host-parasite relationships in CBM by performing an *in vitro* macrophage infection. Peritoneal macrophages were co-cultured with fugal conidia or muriform cells so that macrophage gene expression was assessed after 6 hours of incubation using RNA-Seq. We chose 6 hours as most cytokine and chemokine genes are expressed a few hours after incubation [44]. A larger number of differentially expressed genes (3672 genes) were observed when macrophages were co-cultured with muriform cells, while only 47 genes were differentially expressed in the co-culture with conidia (Figs 3B, S2A and S2B), most of which were downregulated (Fig 3C). Among the 47 genes, 30 of them are common to the 3672 differentially expressed genes listed to the co-culture of macrophages with muriform cells (Fig 3B). Only 3, out of the 30 shared genes, present opposite patterns modulation: *Gm12250*, *Cxcl10* and *Dusp2* (S3 Fig).

Gene Ontology (GO) enrichment analysis of macrophages stimulated with conidia showed few differentially expressed genes correlated to immune response processes (Fig 3C). Differentially expressed (DE) genes detected in co-culture of macrophages with muriform cells included a large amount of upregulated genes related to immune system GO categories, especially those concerning inflammatory response (Fig 3D). The majority of downregulated genes detected in the interaction with muriform cells were annotated as belonging to GO categories related to general metabolic processes (Fig 3D). A variety of processes related to the disease pathogenesis are modulated by fungus so that the full list of ontologies detected to be enriched is listed on S1 and S2 Tables.

Global analysis of DE genes in conidia co-culture showed similar patterns to those found in unstimulated cells (Fig 3A). Conversely, co-culture with muriform cells triggered profound changes in macrophage gene expression profile, featuring gene expression able to promote the establishment of inflammatory response, with intense cell migration and proliferation (S4 Fig). DE genes observed in macrophages stimulated with muriform cells included several genes annotated as involved in inflammatory response (GO:0006954) (S4A Fig). *Il1a*, *Il1b*, *Il1f9*, *Il6*, *Tnf*, *Ptgs2* and *Bdkrb1* were all up regulated in muriform cell co-culture, as well as genes coding to chemokines such as *Cxcl1*, *Ccl2*, *Cxcl2*, *Ccl3*, *Ccl7*, *Cxcl9* and *Cxcl10*, which actively participate in inflammatory processes (S4A Fig). Similarly, most differentially expressed genes related to cell proliferation (S4C Fig), cell migration (S4D Fig) were upregulated when macrophages were co-cultured with muriform cells in contrast to those stimulated with conidia. Muriform cells induced down-regulation of *CCl8*, *Bcl6*, *Tlr1*, *Tlr8*, *Tlr9* and *Il18* (S4A Fig). High cell migration rates are indeed observed after 72h of intraperitoneal inoculation with MC when compared with FC inoculation, confirming that the higher expression of cell migration genes observed in PM-MC interaction is actually related to increased rates of cell migration in the presence muriform cells (S5I Fig).

Other noteworthy genes up-regulated in the interaction with muriform cells included genes coding for important receptors in fungal recognition, such as TLR2, as well as genes coding for co-stimulatory molecules, such as CD40 (S4A Fig). Furthermore, several genes related to angiogenesis and epidermal growth, such as *Ereg*, *Ptgs2* and *Wars* (S4B and S4E Fig) were also up-regulated, which is consistent with common histopathological features observed in human and murine CBM, where great neovascularization is observed. Finally, several pro-apoptotic genes, including *Bcl3*, *Bcl10*, *Malt1* and *Dedd2*, were up-regulated in response to muriform cells infection whereas *Bcl2l12*, coding to anti-apoptotic factor, was down-regulated (S4F Fig), suggesting that infection of macrophages with muriform cells could induce apoptosis.

### Co-culture of macrophages with muriform cells strongly induces proinflammatory cytokines and chemokines, while inhibiting nitric oxide production

**Fig 4:**
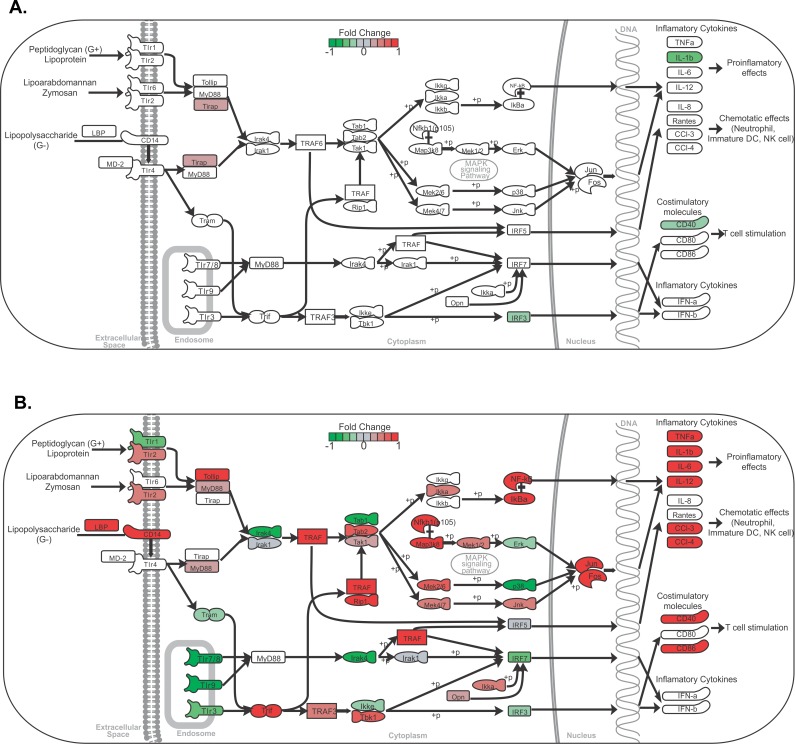
Toll-like receptor signaling pathway overview. Analysis of genes related to the Toll-like receptor signaling pathway showed they were mostly up-regulated in peritoneal macrophage (PM) co-culture with muriform cells (B), but not conidia (A), leading to the expression of pro-inflammatory cytokines and chemokines genes.

**Fig 5:**
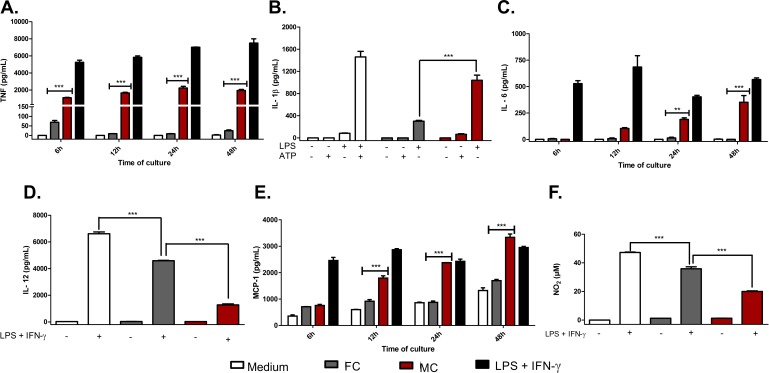
*In vitro* cytokine and chemokine production. The production of cytokines and chemokines by macrophage in conidia (FC) or muriform cell (MC) co-culture supernatants was assessed by ELISA (A-E). NO_2_ concentration in culture supernatants was used as an indicator of NO generation and measured using Griess reagent (F). High levels of TNF-α (A), IL-1β (B) and IL-6 (C) were observed in co-culture with muriform cells, but not with conidia. To assess IL-1β production after 24 hours, peritoneal macrophages required LPS co-stimulation (B). Muriform cells also induced higher levels of MCP-1 after 48h when compared to macrophages infected with conidia (E). Production of IL-12 (D) and NO_2_ (F) was strongly inhibited by muriform cells. **P<0.01 and ***P<0.001.

Gene expression profiling showed the modulation of several genes encoding proinflammatory cytokines and chemokines involved in the Toll-like receptor signaling pathway, which were increased upon interaction with muriform cells (Fig 4B). Interaction with conidia did not show significant changes in gene expression in this pathway except for *Tirap* and *Il1b* gene, which were down-regulated (Fig 4A). The production of cytokines and chemokines involved in the activation of the innate immune response, as well as in the establishment of the inflammatory process, were confirmed by Elisa of co-culture supernatants. Only muriform cells were able to induce TNF-α (Fig 5A) and IL-6 secretion (Fig 5C), with intense MCP-1 production being observed mainly after 48h of co-culture (Fig 5E). Even a higher concentration of conidia (multiplicity of infection of conidia and macrophages, MOI 5:1) was not sufficient to raise the levels of TNF-α and IL-12 (S5A and S5B Fig).

In our in vitro assay model, no fungal morphotype was able to induce the release of IL-1β in its active form without further stimulation. However, both conidia and muriform cells were able to induce IL-1β release in the presence of LPS, with a more intense release observed in macrophages infected with muriform cells (Fig 5B).

Bone marrow-derived macrophages (BMDM) and dendritic cell-derived macrophages (BMDC) were used to confirm that the cytokine profile observed was not restricted to peritoneal macrophages. Similarly, BMDC and BMDM co-cultures with conidia and muriform cells resulted in a higher secretion of proinflammatory cytokines such as TNF-α and IL-1β, without any additional stimulus (S5E–S5H Fig). Nitric oxide (NO) production was inhibited in peritoneal macrophage by fungal forms, especially muriform cells (Fig 5F and S5D Fig). RNA-seq data showed that muriform cells, but not conidia, induced *Il12a* transcript, which encodes for IL12-p35 that forms, with IL-12p40, bioactive IL-12p70 protein (Fig 4A and 4B). Muriform cells and conidia failed to induce the production of IL12p70 in infected macrophages (Fig 5D and S5C Fig).

### *F*. *pedrosoi* conidia phagocytosis does not require FcγR and Dectin-1 recognition

**Fig 6:**
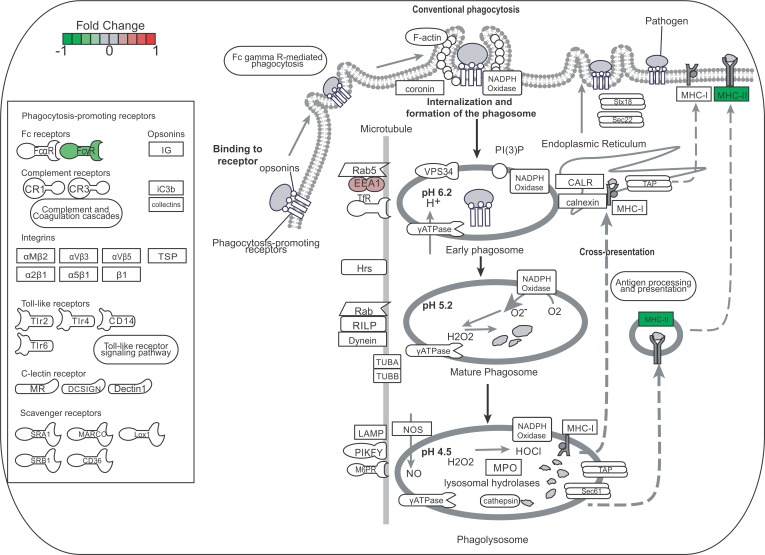
Phagocytosis gene expression in peritoneal macrophages co-culturing with conidia. Peritoneal macrophages (PM) co-culturing with conidia (FC) did not induce much gene expression related to phagocytosis.

**Fig 7:**
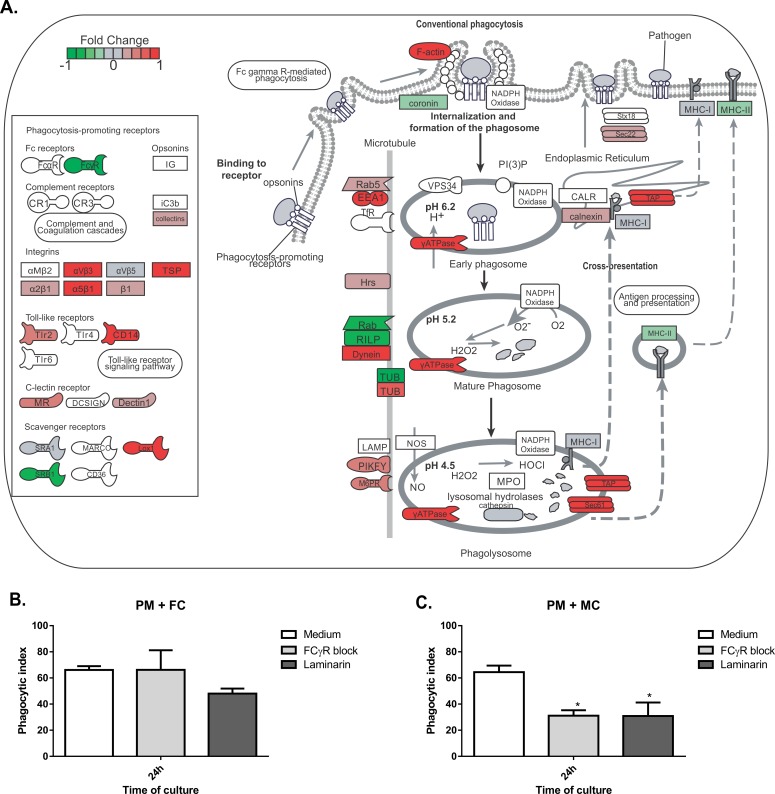
Phagocytosis gene expression in peritoneal macrophages co-culturing with muriform cells and phagocytosis index. Peritoneal macrophages (PM) co-culturing with muriform cells (MC) (A) induced the expression of several genes related to phagocytosis. Muriform cells displayed an elevated expression of Dectin-1 gene while reducing the expression of FcγR gene (A). By blocking Dectin-1 and FcγR with laminarin and FcγR Block, respectively, only muriform cells phagocytosis was impaired (C), while conidia (FC) phagocytosis was not affected (B). *P<0.05, compared to medium control.

*F*. *pedrosoi* conidia are not able to increase the expression of *FcyR* or pattern recognition receptor genes such as *Dectin1* (Fig 6A), even though conidia were internalized after 24h of co-cultivation with peritoneal macrophages (Fig 7B). Macrophages stimulated by the co-culturing with muriform cells showed an elevated expression of Dectin-1 gene while reducing the expression of the FcγR gene (Fig 7A). Only muriform cells phagocytosis was impaired when these membrane receptors were blocked by laminarin and Fcblock, respectively (Fig 7C).

### Intense inflammatory response during murine CBM is correlated with fungus persistence in the host

**Fig 8:**
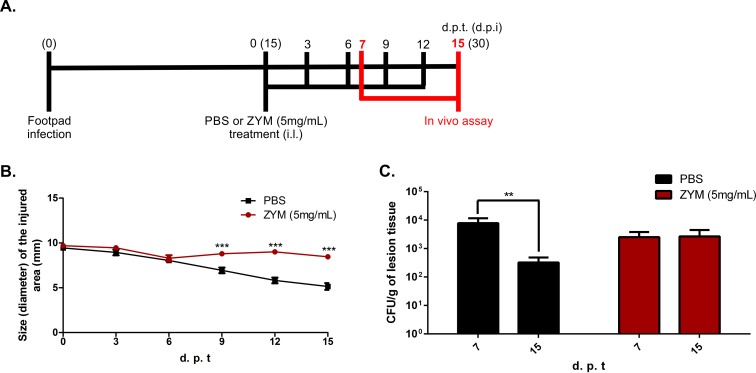
CBM progression in a Zymosan-induced inflammation model. After 15 days post infection with fungal propagules (FP), animals were treated intra lesionally (i.l.) in the footpad with 20μl of a suspension containing 5 mg/ml of zymosan (ZYM) or PBS, until 15 days post treatment start (d.p.t) (A). Animals treated with ZYM displayed intense inflammatory response up to 30 days post infection (d.p.i) (B). Animals facing prolonged inflammation showed no reduction in fungal load over time, as observed for those animals treated with PBS (C). **P<0.01 and ***P<0.001.

Unlike human CBM, which keeps fungal burden and chronic inflammatory processes for long periods of time, all available CBM murine models described to date tend to spontaneously heal after a short period of infection [45]. After 30 days of infection with *F*. *pedrosoi* propagules, it was possible to observe a significant decrease in the inflammatory response followed by a reduction in fungal load. However, it is not clear whether the reduction in the inflammatory response observed in the murine model is the result of the disease’s gradual resolution or the necessary environment for the healing process observed in those animals. We tested the latter hypothesis using a model of chronic inflammation induced by zymosan (ZYM) [25,26]. After 15 days of infection with fungal propagules, animals were treated every three days with 20μl of a suspension containing 5 mg/ml of ZYM (Fig 8A). As expected, those treated with ZYM showed impaired edema reduction in the infected footpad, displaying intense inflammatory response up to 30 days post infection (15 days of treatment) (Fig 8B and S6 Fig). The increase in the inflammatory response impaired the reduction of fungal burden over time (Fig 8C).

## Discussion

Chromoblastomycosis (CBM), together with sporotrichosis and mycetoma, is considered a subcutaneous mycosis of higher incidence in the world [46]. The development of CBM starts as a macular lesion at site of inoculation progressing to a granulomatous inflammatory reaction of cutaneous and subcutaneous tissues. As the initial lesion is asymptomatic, most patients only look for diagnosis during the chronic phase of the disease, with some cases showing edema and bacterial secondary infections that affect the patient’s health and their immune response modulation [47].

In nature, *Fonsecaea pedrosoi* is found in its saprophytic form, and it is not clear whether conidia or hyphae are responsible for initiating the disease. While hyphae and conidia have been described as being present on the surface of the thorns from *Mimosa pudica* [7], muriform cells, similar to those observed in the tissues of patients, are rarely observed in plants [7,48]. Many works were carried out using only condia as the infective form [18,19,49]. Here we demonstrate that hyphae and muriform cells are infective, with both morphotypes being able to establish murine CBM with skin lesions similar to that found in humans, whereas infection with conidia did not reach the chronic phase of the disease. In this model, using immunocompetent mice, inoculated conidia were quickly eliminated from the tissue and few of them turned into muriform cells. Conidia also induced lower levels of inflammatory cytokines secretion in tissue and low induction of transcription *in vitro*.

This low inflammatory response pattern has also been observed after *A*. *fumigatus* spores inhalation, with little neutrophil recruitment and macrophage activation, while the germinating conidia reverse this absent response through β-glucan exposure [50,51]. The absence of β-glucan recognition by dectin-1 is also important for *C*. *albicans* escape from the host’s innate immune response, while dendritic cells discriminate between yeast and hyphae according to β-glucan expression [52]. An alternative explanation for this low innate immune response after infection by conidia is that the small-sized cells can bypass structural tissue defenses, which in turn may go unnoticed by the host immune cells [12]. Nevertheless, despite the lower immune response observed at the inoculation site, *F*. *pedrosoi* conidia has been previously reported to be able to reside inside phagocytes [21,53], which may constitute a survival strategy and could be related to the slow progression of the disease in humans.

Using a different experimental model, Sousa et al [19] have shown that intraperitoneal inoculation of *F*. *pedrosoi* conidia increased IL-10 and decreased TNF-α production by spleen cells. They have also demonstrated that conidia in vitro failed to stimulate macrophages to secrete inflammatory cytokines, such as TNF-α, suggesting that failure in innate recognition can result in chronic infection [19]. However, most CBM lesions in patients present muriform cells in a chronic granulomatous reaction, which is associated with neutrophil-rich, purulent abscesses and a chronic and highly organized inflammatory response coupled with extensive fibrosis [9,20–22]. Our histopathological analysis did not observe conidia in granulomatous reaction during the chronic stage of the infection. Finally, in CBM infected tissue, macrophages (sometimes identified as epithelioid or giant cells) are described as highly activated with prominent expression of proinflammatory cytokines, such as TNF-α [10], corroborating the expression of TNF-a we observed in Figs 2E and 5A. Thus, our data largely suggest a different route for the disease progression according to fungal morphotype, so that the establishment of inflammation is required for successful infection chronicity.

Distinct *A*. *fumigatus* morphotypes were shown to have a complex and distinct response after leukocyte-fungal form interaction, with genes modulated by hyphae being implicated in establishing or prolonging the infection [54]. Thus, understanding how *F*. *pedrosoi* fungal forms are able to modulate the macrophage response is extremely relevant to comprehend the result of the constant interaction of fungal antigens with macrophages at the site of infection and its contribution to disease chronification. Chronic granulomatous infectious diseases are usually characterized by numerous macrophages on lesional tissue so that Sotto et al. have shown the presence of high numbers of macrophages in the skin biopsies of CBM patients [55] and it is common to find muriform cells, sometimes within giant multinucleated cells [6]. In addition, due to high rate of cell migration and proliferation near the infection site, the initial contact of fungus antigens with macrophages and dendritic cells occurs continuously in the course of the disease, even in the later stages.

We observed that macrophages infected in vitro with conidia or muriform cells showed distinct gene expression profiles. Inflammatory response genes, as well as cytokine secretion by macrophages and dendritic cells infected with muriform cells, showed a Th17-inducing differentiation pattern (i.e. enhanced production of IL-6 and IL-1β) concomitant with Th1 suppression (i.e. inhibition of IL-12 production). Muriform cells and conidia failed to induce the production of IL12p70 in infected macrophages (Fig 5D and S4C Fig), suggesting a post-transcriptional and transcriptional negative regulation of il12a by these fungal forms, respectively. In fact, conidia of several species of *Fonsecae* disrupt nuclear IRF1 activity, which is crucial for il12a transcription via nucleosome remodeling [56]. Furthermore, both fungal forms inhibit the production of IL-12p70 by LPS-treated macrophages (Fig 5D). In this context, the low production or the absence of functional IL-12 may restrict the generation of Th1 immunity leading to the suppression of macrophage fungicidal activity.

Microorganism size and shape has been correlated with virulence and morphogenesis [57], and cell size can interfere in multiple host immune responses, such as phagocytosis. Small structures can disperse easily through the tissue, as described for *Cryptococcus* spores [58]. *F*. *pedrosoi* conidia have 1.5 x 3 μm in diameter [7], which can be easily internalized by macrophages. Although *F*. *pedrosoi* phagocytosis mechanisms are not completely understood, it is known that neutrophils, macrophages, dendritic cells and Langherhan cells are able to internalize the fungus [53,59,60]. The interaction between Langherhan cells and conidia, but not muriform cells, yields a decrease in the expression of CD40 and B7-2, impairing antigen presentation and adaptive immune response [59]. Also, the fungal clearance by phagocytes is dependent on the interaction between fungal pathogen-associated molecular patterns (PAMPs) and phagocytic receptors [61].

Our results show that recognition by Dectin-1 or FCγR is important only to muriform cells internalization, showing that conidia and muriform cells internalization processes occur through distinct recognition receptors. This discriminated recognition was described for *S*. *schenckii* morphotypes, in which phagocytosis of conidia failed to induce a pro-inflammatory response and favored the fungus’s survival [62]. In classical receptor-mediated phagocytosis, the phagocytic receptors-PAMP interaction is not only important for fungal engulfment and killing, but also throughout the phagosome maturation [61]. Therefore, internalized conidia without phagocytic receptor interaction can remain silent inside macrophages without activating the cell, as well as the host immune system, through cytokines production and inflammasome activation. Alternatively, other receptor-independent mechanisms for microorganism uptake, such as clathrin-mediated endocytosis, macropinocytosis and lipidraft/caveolae-dependent endocytosis [63], can be used in the response to certain fungi, such as *C*. *neoformans* [64].

This scenario may contribute to the induction and establishment of the chronic inflammatory response observed in CBM characterized by dense inflammatory infiltrates that are rich in granulocytes, especially neutrophils, along with macrophages showing poor fungicidal activity against muriform cells.

We also observed several genes linked to NFκB activation, including those associated with apoptosis process. Apoptosis is an important component of protective immunity against *Histoplasma capsulatum* and *P*. *brasiliensis*, and they have been shown to be associated with outcomes of infection [65,66]. In our model, apoptotic genes were up-regulated by muriform cells and our group has reported similar results in an analysis of the transcriptional response of peritoneal macrophages [67] and dendritic cells [68] to *P*. *brasiliensis* infection in vitro. It is reasonable to presume that despite the induction of apoptosis, phagocytes do not promptly remove apoptotic cells and necrosis can start, increasing the inflammation [69].

Early inflammation is beneficial in containing the infection, but an uncontrolled inflammatory response is detrimental and may eventually oppose disease eradication, being evident in mice with chronic granulomatous disease [2]. Animals infected with fungal propagules and later treated with high doses of Zymozan showed no reduction in fungal burden over time, confirming the detrimental nature of an intense inflammatory response in CBM, which may contribute to a chronic infection state. Besides, previous work showed that modulation of the inflammatory response mediated by DNA-hsp65 vaccine was able to accelerate the healing process of experimental CBM [23].

Altogether, our results show that muriform cells are able to induce inflammatory response in the course of murine CBM, allowing the fungus to persist in the host and involving inflammation as an important factor in CBM chronicity. In this way, CBM chronicity may not be due to a failure in inducing an inflammatory response, but rather be related to the host's inability to regulate the exacerbated inflammatory response induced by muriform cells. With this in mind, new therapeutic approaches concerning the modulation of inflammatory response can be developed, allowing reduced chemotherapy periods and lowering relapse rates.

## Supporting information

S1 Fig Skin lesions analysis in mice infected with *F. pedrosoi* fungal forms. Macroscopic aspect of the disease (A) and histopathological analyses (B) showed that infection with hyphae (FH) or muriform cells (MC), but not with conidia (FC), is capable of inducing skin lesions similar to that observed in humans with CBM. Ulcerative lesions similar to those found in humans arose after 15 days in all infected animals, but not in those infected with conidia. After 30 days of infection, progressive healing in the injured area was evident in all groups. After 45 days only animals infected with MCs still showed significant edema, in contrast to all others, which presented similar features to those found in uninfected animals (A). Histopathological analysis showed neutrophilic and histiocytic inflammatory infiltrate in the first 3 days of infection with all fungal forms. Ulceration of exudative areas, with the presence of necrotic material and fungal cells, as well as a multifocal lymphocytic infiltrate outlining a granulomatous lesion aspect was observed after 15 days of infection with all fungal forms except conidia (B). After 30 days of infection, an intense tissue repair was already observed in animals infected with conidia, while for those infected with hyphae and fungal propagules (FP), an intense healing process was only seen at 45 days post-infection, with the presence of fibroblasts and collagen deposition. At that time, only animals infected with muriform cells still exhibited exudative areas (B).
(TIF)

S2 Fig Identification of differentially expressed genes. Funnel chart (A) and VennEuler diagram (B) displaying differentially expressed genes when False Discovery Rates (FDR) < 0.05 and Fold Change cutoff (FC cutoff) > 1.4, respectively.
(TIF)

S3 Fig Common differentially expressed genes to PM-FC and PM-MC interaction. Heatmap of 30 differentially expressed genes in peritoneal macrophages (PM) infected with conidia (FC) or muriform cells (MC). Heatmap was build based on fold-change values.
(TIF)

S4 Fig Cell differentiation GO enrichment analysis. Heatmap of differentially expressed genes in peritoneal macrophages (PM) infected with conidia (FC) or muriform cells (MC) correlated to inflammatory response (GO: 0006954), angiogenesis (GO:0001525), cell proliferation (GO:0008283), cell migration (GO:0016477), regulation of angiogenesis (GO: 0045765) and regulation of apoptotic process (GO:0042981).
(TIF)

S5 Fig Cell migration and cytokine production after incubation with conidia or muriform cells using distinct phagocytes and MOI. TNF-α (A) and IL-6 (B) production are not increased in higher concentration of conidia (MOI 5:1 of conidia and peritoneal macrophage, respectively). IL-12 (C) and NO2 (D) were not detected after 6, 12, 24 or 48 hours of PM incubation with FC or MCs. Fungal cells co-culture with mouse bone marrow-derived macrophages (BMDMs) and dendritic cells (BMDCs) showed similar patterns of TNF-α (E-F) and IL-1β (G-H) production compared to PM cells after 24 hours. Further stimulation was not required for IL-1β production in BMDM-MC (G) or BMDC-MC (H) co-culture. Peritoneal inoculation with 10^6^ cells of each fungal form revealed intense cell migration to peritoneal cavity induced by muriform cells compared to conidia (FC) inoculation (I). ***P<0.001, **P<0.01.
(TIF)

S6 Fig Skin lesions analysis in mice infected with *F. pedrosoi* and treated with zymosan. After 15 days post infection with FP, animals were treated intra lesionally (i.l.) in the footpad with 20μl of a suspension containing 5 mg/ml of zymosan (ZYM) or PBS, until 15 days post treatment start (d.p.t). DPI and HE or Masson’s trichrome stain are indicated in the figure.
(TIF)

S1 Table Gene ontology enrichment results for biological process categories in macrophage co-culture with *F. pedrosoi* conidia. (PDF)

S2 Table Gene ontology enrichment results for biological process categories in macrophage co-culture with muriform cells. (PDF)
