# Supplementary figures and images for: Modulation of the immune response by Fonsecaea pedrosoi morphotypes in the course of experimental chromoblastomycosis and their role on inflammatory response chronicity
Source: PLoS Negl Trop Dis. 2017 Mar 29;11(3):e0005461. doi: 10.1371/journal.pntd.0005461 (PMC5391973; doi:10.1371/journal.pntd.0005461)

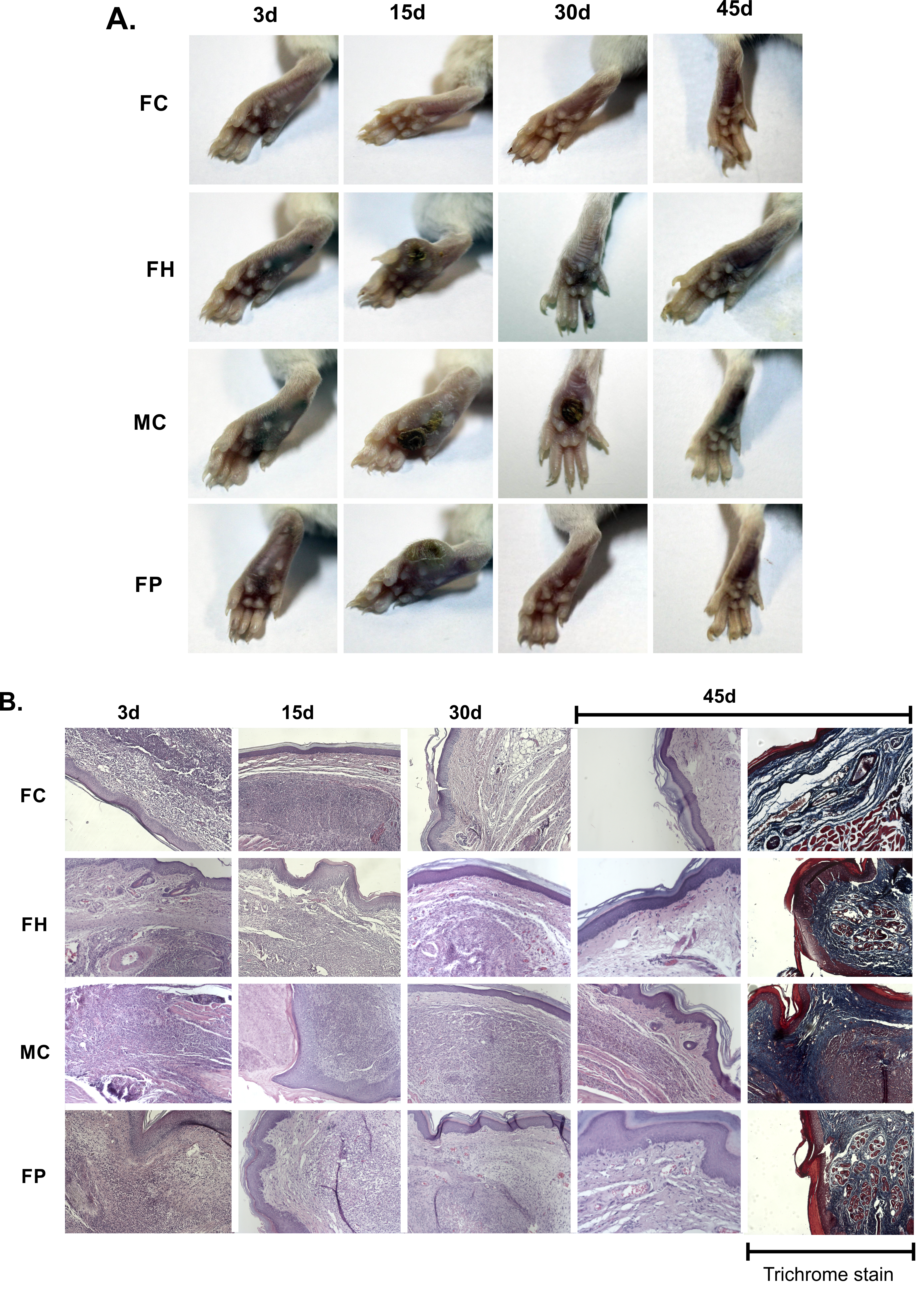

Supplement: S1 Fig — Macroscopic aspect of the disease (A) and histopathological analyses (B) showed that infection with hyphae (FH) or muriform cells (MC), but not with conidia (FC), is capable of inducing skin lesions similar to that observed in humans with CBM. Ulcerative lesions similar to those found in humans arose after 15 days in all infected animals, but not in those infected with conidia. After 30 days of infection, progressive healing in the injured area was evident in all groups. After 45 days only animals infected with MCs still showed significant edema, in contrast to all others, which presented similar features to those found in uninfected animals (A). Histopathological analysis showed neutrophilic and histiocytic inflammatory infiltrate in the first 3 days of infection with all fungal forms. Ulceration of exudative areas, with the presence of necrotic material and fungal cells, as well as a multifocal lymphocytic infiltrate outlining a granulomatous lesion aspect was observed after 15 days of infection with all fungal forms except conidia (B). After 30 days of infection, an intense tissue repair was already observed in animals infected with conidia, while for those infected with hyphae and fungal propagules (FP), an intense healing process was only seen at 45 days post-infection, with the presence of fibroblasts and collagen deposition. At that time, only animals infected with muriform cells still exhibited exudative areas (B). (TIF) [file pntd.0005461.s001.tif]

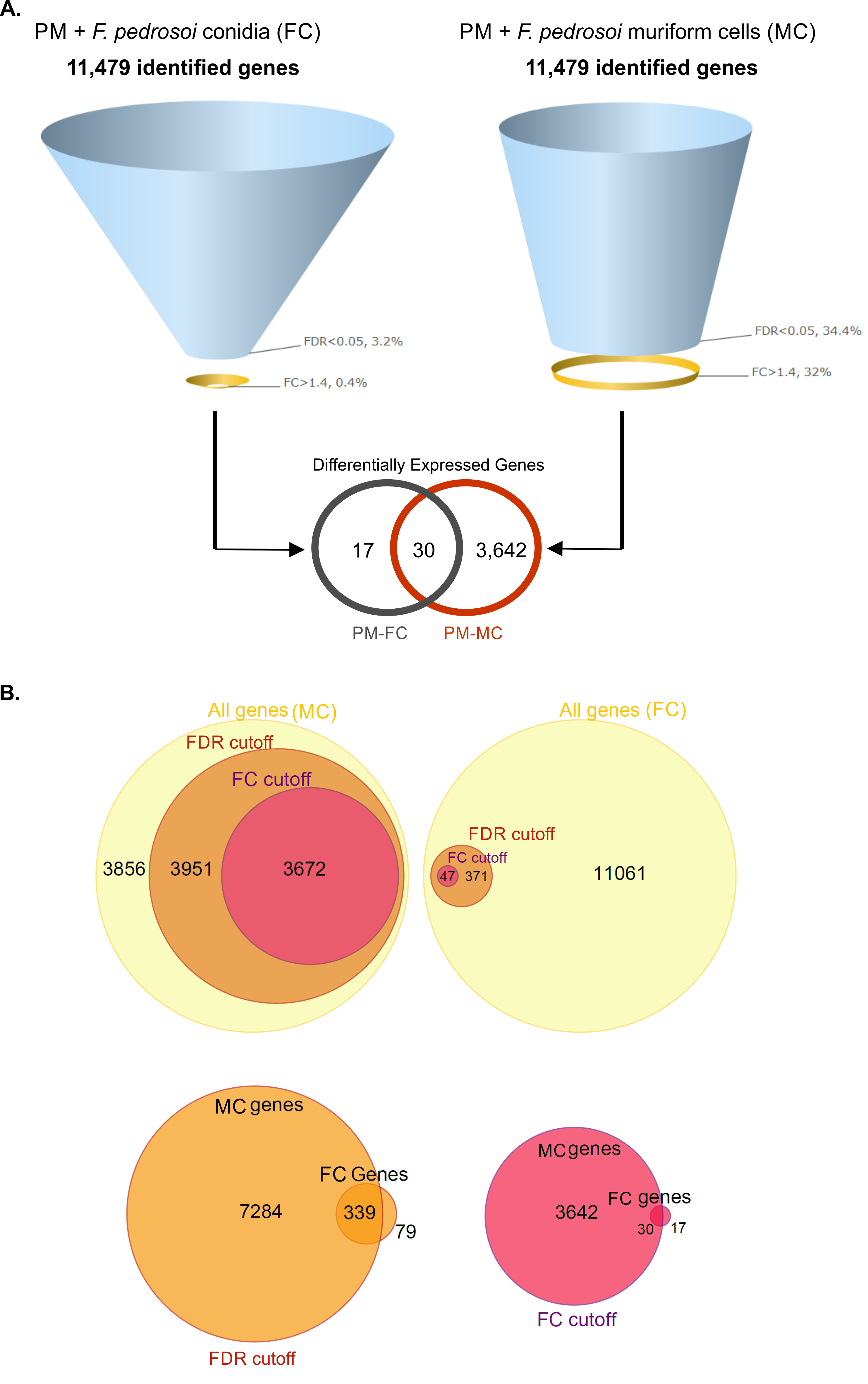

Supplement: S2 Fig — Funnel chart (A) and VennEuler diagram (B) displaying differentially expressed genes when False Discovery Rates (FDR) < 0.05 and Fold Change cutoff (FC cutoff) > 1.4, respectively. (TIF) [file pntd.0005461.s002.tif]

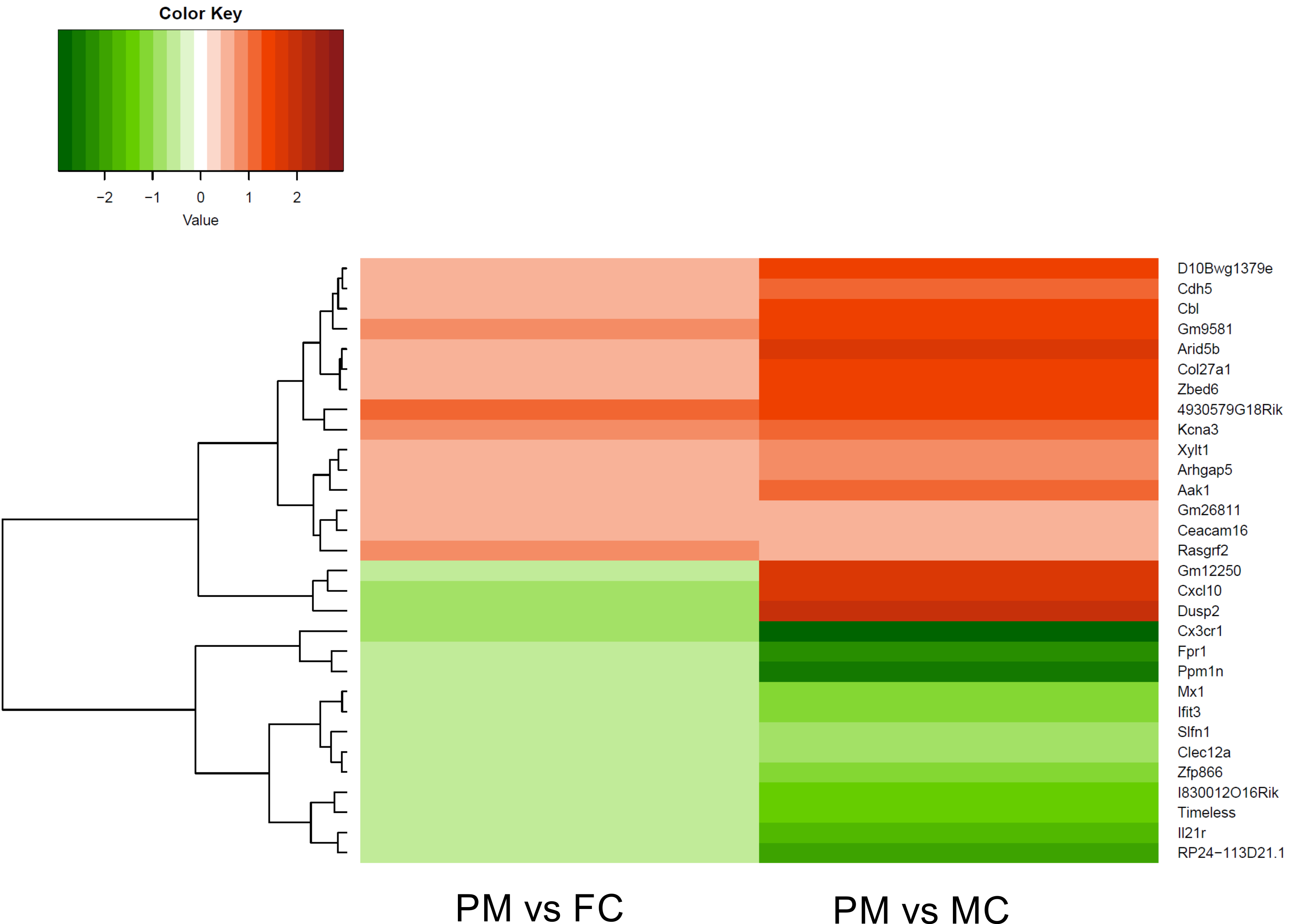

Supplement: S3 Fig — Heatmap of 30 differentially expressed genes in peritoneal macrophages (PM) infected with conidia (FC) or muriform cells (MC). Heatmap was build based on fold-change values. (TIF) [file pntd.0005461.s003.tif]

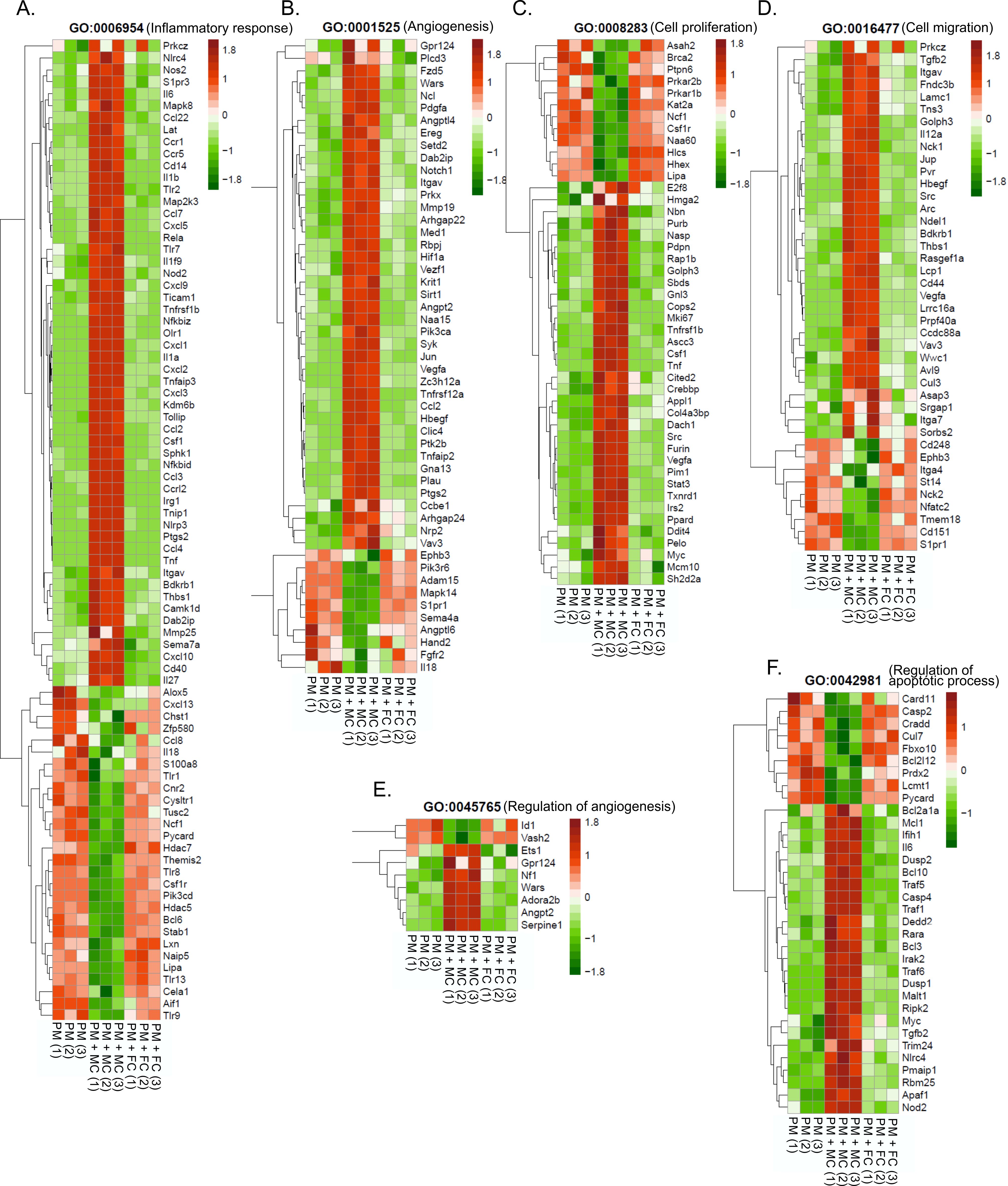

Supplement: S4 Fig — Heatmap of differentially expressed genes in peritoneal macrophages (PM) infected with conidia (FC) or muriform cells (MC) correlated to inflammatory response (GO: 0006954), angiogenesis (GO:0001525), cell proliferation (GO:0008283), cell migration (GO:0016477), regulation of angiogenesis (GO: 0045765) and regulation of apoptotic process (GO:0042981). (TIF) [file pntd.0005461.s004.tif]

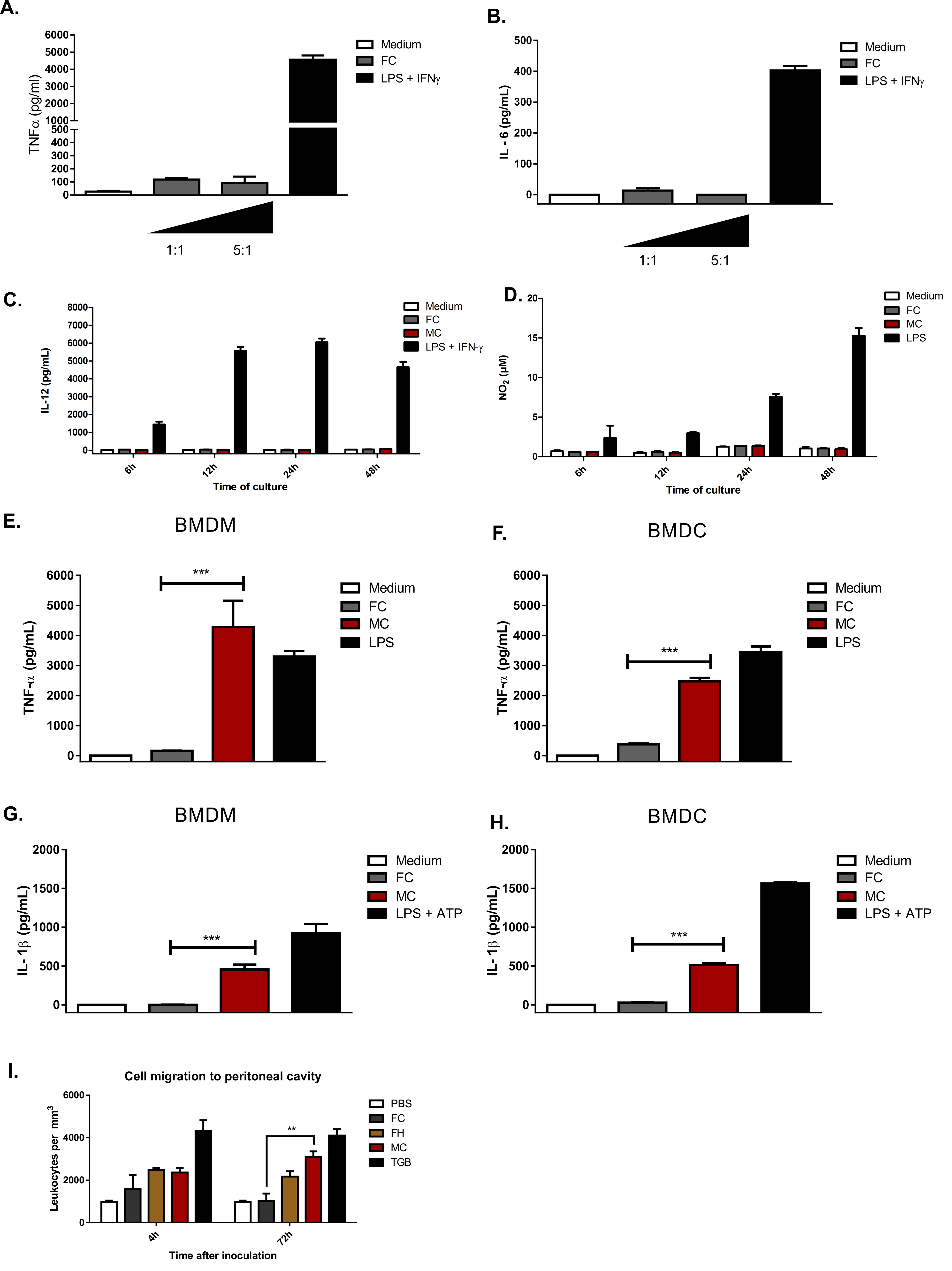

Supplement: S5 Fig — TNF-α (A) and IL-6 (B) production are not increased in higher concentration of conidia (MOI 5:1 of conidia and peritoneal macrophage, respectively). IL-12 (C) and NO2 (D) were not detected after 6, 12, 24 or 48 hours of PM incubation with FC or MCs. Fungal cells co-culture with mouse bone marrow-derived macrophages (BMDMs) and dendritic cells (BMDCs) showed similar patterns of TNF-α (E-F) and IL-1β (G-H) production compared to PM cells after 24 hours. Further stimulation was not required for IL-1β production in BMDM-MC (G) or BMDC-MC (H) co-culture. Peritoneal inoculation with 106 cells of each fungal form revealed intense cell migration to peritoneal cavity induced by muriform cells compared to conidia (FC) inoculation (I). ***P<0.001, **P<0.01. (TIF) [file pntd.0005461.s005.tif]

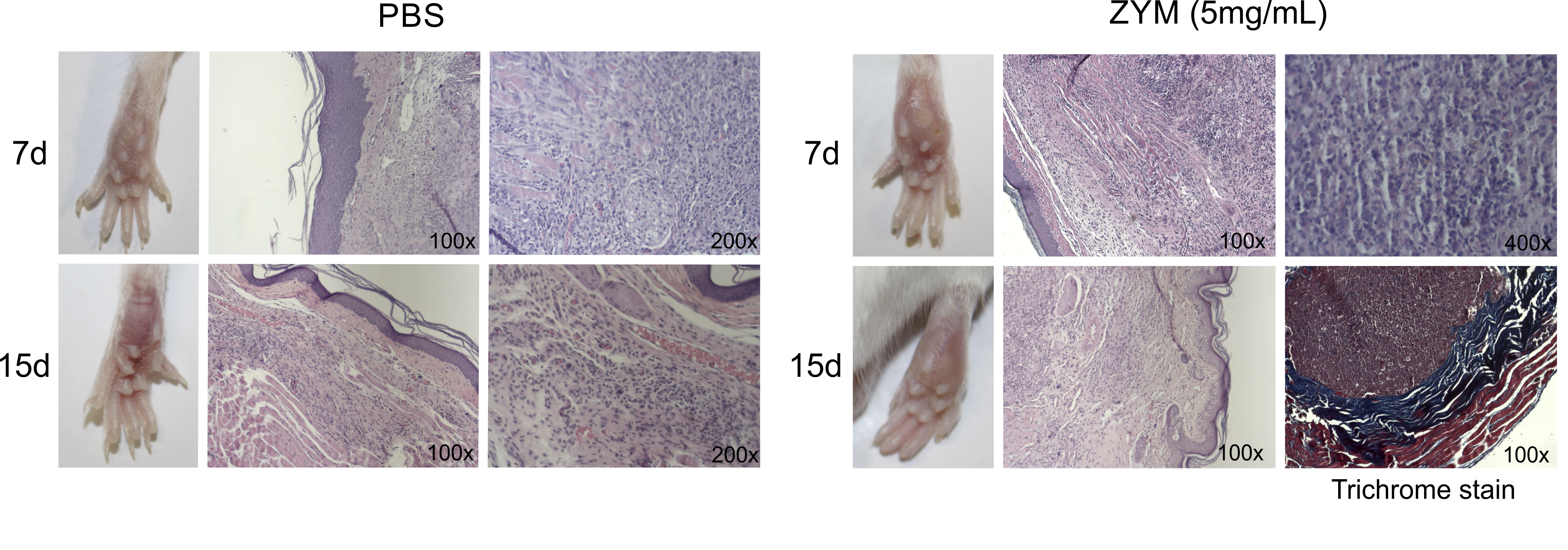

Supplement: S6 Fig — After 15 days post infection with FP, animals were treated intra lesionally (i.l.) in the footpad with 20μl of a suspension containing 5 mg/ml of zymosan (ZYM) or PBS, until 15 days post treatment start (d.p.t). DPI and HE or Masson’s trichrome stain are indicated in the figure. (TIF) [file pntd.0005461.s006.tif]
