# Supplementary material for: Modulation of the immune response by Fonsecaea pedrosoi morphotypes in the course of experimental chromoblastomycosis and their role on inflammatory response chronicity
Source: PLoS Negl Trop Dis. 2017 Mar 29;11(3):e0005461. doi: 10.1371/journal.pntd.0005461 (PMC5391973; doi:10.1371/journal.pntd.0005461)
Supplement: S1 Table — (PDF) [file pntd.0005461.s007.pdf]

Supporting Table 1: gene ontology enrichment results for biological process categories in macrophage co-culture with *F. pedrosoi* conidia.

#	GO.ID	Term	Annotated	Significant	Expected	Raw.pValue	FDR.corrected
1	GO:0006955	immune response	576	12	2.21	9.9e-07	0.0040739
2	GO:0002376	immune system process	1159	16	4.44	2.4e-06	0.004938
3	GO:0098542	defense response to other organism	196	6	0.75	9.2e-05	0.1261933
4	GO:0002252	immune effector process	325	7	1.24	0.00021	0.2160375
5	GO:0051607	defense response to virus	109	4	0.42	0.00077	0.6103917
6	GO:0034097	response to cytokine	299	6	1.15	0.00089	0.6103917
7	GO:0009615	response to virus	135	4	0.52	0.00171	0.7893318
8	GO:0070098	chemokine-mediated signaling pathway	17	2	0.07	0.00188	0.7893318
9	GO:0043207	response to external biotic stimulus	353	6	1.35	0.00209	0.7893318
10	GO:0051707	response to other organism	353	6	1.35	0.00209	0.7893318
11	GO:0071345	cellular response to cytokine stimulus	240	5	0.92	0.00211	0.7893318
12	GO:0002455	humoral immune response mediated by circ...	20	2	0.08	0.00260	0.8261654
13	GO:0009607	response to biotic stimulus	369	6	1.41	0.00261	0.8261654
14	GO:0009605	response to external stimulus	888	9	3.4	0.00555	1
15	GO:0002682	regulation of immune system process	582	7	2.23	0.00606	1
16	GO:0050896	response to stimulus	3862	23	14.79	0.00691	1
17	GO:0050853	B cell receptor signaling pathway	33	2	0.13	0.00701	1
18	GO:0030100	regulation of endocytosis	112	3	0.43	0.00890	1
19	GO:0016525	negative regulation of angiogenesis	41	2	0.16	0.01068	1
20	GO:0006959	humoral immune response	49	2	0.19	0.01502	1
21	GO:0007166	cell surface receptor signaling pathway	1261	10	4.83	0.01819	1
22	GO:0042113	B cell activation	147	3	0.56	0.01848	1
23	GO:0002683	negative regulation of immune system pro...	148	3	0.57	0.01881	1
24	GO:0019221	cytokine-mediated signaling pathway	149	3	0.57	0.01915	1
25	GO:0042100	B cell proliferation	56	2	0.21	0.01934	1
26	GO:0006952	defense response	570	6	2.18	0.02038	1
27	GO:0002250	adaptive immune response	153	3	0.59	0.02053	1
28	GO:0009266	response to temperature stimulus	60	2	0.23	0.02202	1
29	GO:0045321	leukocyte activation	428	5	1.64	0.02289	1
30	GO:0046651	lymphocyte proliferation	160	3	0.61	0.02307	1
31	GO:0032943	mononuclear cell proliferation	161	3	0.62	0.02345	1
32	GO:0016064	immunoglobulin mediated immune response	63	2	0.24	0.02412	1
33	GO:0070661	leukocyte proliferation	164	3	0.63	0.02460	1
34	GO:0019724	B cell mediated immunity	65	2	0.25	0.02557	1
35	GO:0030183	B cell differentiation	67	2	0.26	0.02705	1
36	GO:0050851	antigen receptor-mediated signaling path...	70	2	0.27	0.02933	1
37	GO:0050776	regulation of immune response	312	4	1.19	0.03075	1
38	GO:0019882	antigen processing and presentation	77	2	0.29	0.03495	1
39	GO:0002429	immune response-activating cell surface ...	80	2	0.31	0.03748	1
40	GO:0002251	organ or tissue specific immune response	10	1	0.04	0.03766	1
41	GO:0003416	endochondral bone growth	10	1	0.04	0.03766	1
42	GO:0042090	interleukin-12 biosynthetic process	10	1	0.04	0.03766	1
43	GO:0045075	regulation of interleukin-12 biosyntheti...	10	1	0.04	0.03766	1

44	GO:0070570	regulation of neuron projection regenera...	10	1	0.04	0.03766	1
45	GO:2000114	regulation of establishment of cell pola...	10	1	0.04	0.03766	1
46	GO:0060627	regulation of vesicle-mediated transport	195	3	0.75	0.03825	1
47	GO:0001775	cell activation	491	5	1.88	0.03827	1
48	GO:0009409	response to cold	11	1	0.04	0.04135	1
49	GO:0034114	regulation of heterotypic cell-cell adhe...	11	1	0.04	0.04135	1
50	GO:0072583	clathrin-mediated endocytosis	11	1	0.04	0.04135	1
