# Supplementary material for: Modulation of the immune response by Fonsecaea pedrosoi morphotypes in the course of experimental chromoblastomycosis and their role on inflammatory response chronicity
Source: PLoS Negl Trop Dis. 2017 Mar 29;11(3):e0005461. doi: 10.1371/journal.pntd.0005461 (PMC5391973; doi:10.1371/journal.pntd.0005461)
Supplement: S2 Table — (PDF) [file pntd.0005461.s008.pdf]

Supporting Table 2: gene ontology enrichment results for biological process categories in macrophage co-culture with muriform cells.

#	GO.ID	Term	Annotated	Significant	Expected	Raw.pValue	FDR,corrected
1	GO:0002376	immune system process	1159	479	371.14	1.0e-12	5.90E-06
2	GO:0006954	inflammatory response	284	146	90.94	5.5e-12	1.62E-03
3	GO:0006955	immune response	576	260	184.45	9.4e-12	1.85E-03
4	GO:0006952	defense response	570	251	182.53	4.6e-10	6.78E-03
5	GO:0048534	hematopoietic or lymphoid organ developm...	507	225	162.35	1.7e-09	2.01E-01
6	GO:0001816	cytokine production	341	161	109.2	2.1e-09	2.06E-02
7	GO:0001775	cell activation	491	218	157.23	2.9e-09	2.42E+08
8	GO:0002520	immune system development	537	235	171.96	3.4e-09	2.42E+08
9	GO:0044707	single-multicellular organism process	2888	1050	924.81	3.7e-09	2.42E+08
10	GO:0032501	multicellular organismal process	2956	1070	946.58	7.6e-09	4.48E-01
11	GO:0045321	leukocyte activation	428	192	137.06	1.0e-08	4.92E-03
12	GO:0051240	positive regulation of multicellular org...	337	157	107.92	1.0e-08	4.92E-03
13	GO:0002682	regulation of immune system process	582	249	186.37	1.4e-08	6.35E+08
14	GO:0030097	hemopoiesis	488	213	156.27	2.4e-08	1.01E+09
15	GO:0023052	signaling	2648	962	847.95	3.1e-08	1.14E+02
16	GO:0044700	single organism signaling	2648	962	847.95	3.1e-08	1.14E+02
17	GO:0051239	regulation of multicellular organismal p...	1257	488	402.52	3.4e-08	1.18E-01
18	GO:0007165	signal transduction	2460	898	787.75	4.1e-08	1.34E+09
19	GO:0002684	positive regulation of immune system pro...	359	162	114.96	9.0e-08	2.79E+09
20	GO:0001817	regulation of cytokine production	302	140	96.71	9.6e-08	2.83E+00
21	GO:0002521	leukocyte differentiation	300	139	96.07	1.1e-07	3.09E+09
22	GO:0007154	cell communication	2715	979	869.41	1.2e-07	3.22E+09
23	GO:0002694	regulation of leukocyte activation	242	116	77.49	1.4e-07	3.59E+09
24	GO:0050865	regulation of cell activation	260	123	83.26	1.5e-07	3.69E+00
25	GO:0023051	regulation of signaling	1604	601	513.64	3.0e-07	7.08E-01
26	GO:0010646	regulation of cell communication	1609	602	515.24	3.7e-07	8.30E+09
27	GO:0042127	regulation of cell proliferation	779	313	249.45	3.8e-07	8.30E+09
28	GO:0001819	positive regulation of cytokine producti...	162	82	51.88	5.8e-07	1.22E-04
29	GO:0040011	locomotion	744	299	238.25	7.0e-07	1.36E-04
30	GO:1902107	positive regulation of leukocyte differe...	89	51	28.5	7.0e-07	1.36E-04
31	GO:0009605	response to external stimulus	888	350	284.36	7.2e-07	1.36E-04
32	GO:0016477	cell migration	608	250	194.7	7.4e-07	1.36E-04
33	GO:0001562	response to protozoan	23	19	7.37	8.1e-07	1.45E-04
34	GO:0008283	cell proliferation	982	382	314.46	1.0e-06	1.73E-04
35	GO:0007275	multicellular organismal development	2314	836	741	1.2e-06	2.02E-04
36	GO:0035556	intracellular signal transduction	1455	545	465.93	1.3e-06	2.13E-04
37	GO:1902531	regulation of intracellular signal trans...	957	372	306.45	1.6e-06	2.55E-04
38	GO:0051046	regulation of secretion	288	130	92.22	1.7e-06	2.57E-04
39	GO:0070372	regulation of ERK1 and ERK2 cascade	112	60	35.87	1.7e-06	2.57E-04
40	GO:0008284	positive regulation of cell proliferatio...	422	180	135.13	1.9e-06	2.80E-04
41	GO:0042742	defense response to bacterium	89	50	28.5	2.0e-06	2.81E-04
42	GO:0009966	regulation of signal transduction	1477	551	472.97	2.0e-06	2.81E-04
43	GO:0046649	lymphocyte activation	366	159	117.2	2.1e-06	2.81E-04

44	GO:0048513	organ development	1498	558	479.7	2.1e-06	2.81E-04
45	GO:0051241	negative regulation of multicellular org...	209	99	66.93	2.2e-06	2.88E-04
46	GO:0009888	tissue development	884	345	283.08	2.6e-06	3.33E-04
47	GO:0070371	ERK1 and ERK2 cascade	118	62	37.79	2.8e-06	3.37E-04
48	GO:0048870	cell motility	642	259	205.58	2.8e-06	3.37E-04
49	GO:0051674	localization of cell	642	259	205.58	2.8e-06	3.37E-04
50	GO:0032879	regulation of localization	1094	418	350.33	2.9e-06	3.42E-04
51	GO:0070663	regulation of leukocyte proliferation	123	64	39.39	3.0e-06	3.47E-04
52	GO:0060341	regulation of cellular localization	532	219	170.36	3.3e-06	3.74E-04
53	GO:0032944	regulation of mononuclear cell prolifera...	121	63	38.75	3.5e-06	3.89E-04
54	GO:0050896	response to stimulus	3862	1341	1236.71	4.0e-06	4.37E-04
55	GO:0045639	positive regulation of myeloid cell diff...	59	36	18.89	4.2e-06	4.50E-04
56	GO:0002763	positive regulation of myeloid leukocyte...	38	26	12.17	4.4e-06	4.63E-04
57	GO:0048518	positive regulation of biological proces...	2593	923	830.34	4.7e-06	4.86E-04
58	GO:0044767	single-organism developmental process	2733	969	875.17	5.2e-06	5.22E-04
59	GO:0051249	regulation of lymphocyte activation	215	100	68.85	5.4e-06	5.22E-04
60	GO:0009306	protein secretion	132	67	42.27	5.4e-06	5.22E-04
61	GO:0042832	defense response to protozoan	21	17	6.72	5.4e-06	5.22E-04
62	GO:0050670	regulation of lymphocyte proliferation	120	62	38.43	5.7e-06	5.42E-04
63	GO:0051049	regulation of transport	754	296	241.45	8.1e-06	7.58E-04
64	GO:0031349	positive regulation of defense response	136	68	43.55	9.0e-06	8.29E-04
65	GO:0042116	macrophage activation	33	23	10.57	9.8e-06	8.85E-04
66	GO:0048583	regulation of response to stimulus	1828	664	585.37	9.9e-06	8.85E-04
67	GO:0048519	negative regulation of biological proces...	2358	841	755.09	1.1e-05	9.54E-04
68	GO:0070665	positive regulation of leukocyte prolife...	79	44	25.3	1.1e-05	9.54E-04
69	GO:0048522	positive regulation of cellular process	2353	839	753.49	1.2e-05	1.03E-03
70	GO:0032946	positive regulation of mononuclear cell ...	77	43	24.66	1.3e-05	1.10E-03
71	GO:0045595	regulation of cell differentiation	781	304	250.1	1.4e-05	1.15E-03
72	GO:0048731	system development	2020	727	646.85	1.4e-05	1.15E-03
73	GO:0032940	secretion by cell	404	169	129.37	1.5e-05	1.21E-03
74	GO:0032502	developmental process	2752	970	881.26	1.6e-05	1.28E-03
75	GO:0050663	cytokine secretion	80	44	25.62	1.7e-05	1.34E-03
76	GO:0030098	lymphocyte differentiation	194	90	62.12	1.8e-05	1.40E-03
77	GO:0048869	cellular developmental process	1946	701	623.16	1.9e-05	1.40E-03
78	GO:0001568	blood vessel development	348	148	111.44	1.9e-05	1.40E-03
79	GO:0001944	vasculature development	367	155	117.52	1.9e-05	1.40E-03
80	GO:0050863	regulation of T cell activation	146	71	46.75	1.9e-05	1.40E-03
81	GO:0048523	negative regulation of cellular process	2174	777	696.17	2.0e-05	1.44E-03
82	GO:0050794	regulation of cellular process	4989	1697	1597.6	2.0e-05	1.44E-03
83	GO:0050867	positive regulation of cell activation	154	74	49.31	2.2e-05	1.56E-03
84	GO:0009617	response to bacterium	221	100	70.77	2.3e-05	1.58E-03
85	GO:0050671	positive regulation of lymphocyte prolif...	76	42	24.34	2.3e-05	1.58E-03
86	GO:0046903	secretion	456	187	146.02	2.3e-05	1.58E-03
87	GO:0051716	cellular response to stimulus	3156	1101	1010.63	2.4e-05	1.63E-03
88	GO:0051047	positive regulation of secretion	162	77	51.88	2.5e-05	1.68E-03
89	GO:0050793	regulation of developmental process	1089	409	348.72	2.6e-05	1.69E-03
90	GO:0072358	cardiovascular system development	529	213	169.4	2.6e-05	1.69E-03
91	GO:0072359	circulatory system development	529	213	169.4	2.6e-05	1.69E-03

92	GO:0048584	positive regulation of response to stimu...	910	347	291.4	2.7e-05	1.71E-03
93	GO:0046883	regulation of hormone secretion	93	49	29.78	2.7e-05	1.71E-03
94	GO:0031347	regulation of defense response	251	111	80.38	2.8e-05	1.76E-03
95	GO:0002274	myeloid leukocyte activation	98	51	31.38	2.9e-05	1.80E-03
96	GO:0042107	cytokine metabolic process	72	40	23.06	3.0e-05	1.84E-03
97	GO:0030154	cell differentiation	1792	647	573.84	3.2e-05	1.93E-03
98	GO:0050789	regulation of biological process	5262	1782	1685.02	3.2e-05	1.93E-03
99	GO:0042110	T cell activation	247	109	79.1	3.7e-05	2.20E-03
100	GO:0030334	regulation of cell migration	352	148	112.72	3.8e-05	2.24E-03
101	GO:0003008	system process	560	223	179.33	3.9e-05	2.28E-03
102	GO:0070661	leukocyte proliferation	164	77	52.52	4.2e-05	2.41E-03
103	GO:0051270	regulation of cellular component movemen...	402	166	128.73	4.2e-05	2.41E-03
104	GO:0043367	CD4-positive, alpha-beta T cell differen...	35	23	11.21	4.3e-05	2.44E-03
105	GO:0000122	negative regulation of transcription fro...	416	171	133.21	4.4e-05	2.47E-03
106	GO:0044057	regulation of system process	185	85	59.24	4.6e-05	2.56E-03
107	GO:2000026	regulation of multicellular organismal d...	812	311	260.02	4.8e-05	2.65E-03
108	GO:1903034	regulation of response to wounding	193	88	61.8	4.9e-05	2.68E-03
109	GO:0042089	cytokine biosynthetic process	71	39	22.74	5.3e-05	2.87E-03
110	GO:0009893	positive regulation of metabolic process	1511	550	483.86	5.4e-05	2.90E-03
111	GO:0019220	regulation of phosphate metabolic proces...	1065	398	341.04	5.5e-05	2.92E-03
112	GO:0033002	muscle cell proliferation	95	49	30.42	5.6e-05	2.95E-03
113	GO:0042108	positive regulation of cytokine biosynth...	44	27	14.09	5.7e-05	2.98E-03
114	GO:0042035	regulation of cytokine biosynthetic proc...	62	35	19.85	5.9e-05	3.05E-03
115	GO:0065007	biological regulation	5464	1843	1749.7	6.0e-05	3.08E-03
116	GO:0051174	regulation of phosphorus metabolic proce...	1069	399	342.32	6.2e-05	3.13E-03
117	GO:1902533	positive regulation of intracellular sig...	446	181	142.82	6.2e-05	3.13E-03
118	GO:2000145	regulation of cell motility	369	153	118.16	6.5e-05	3.25E-03
119	GO:0023056	positive regulation of signaling	709	274	227.04	6.7e-05	3.32E-03
120	GO:0045597	positive regulation of cell differentiat...	372	154	119.12	6.8e-05	3.34E-03
121	GO:0002696	positive regulation of leukocyte activat...	148	70	47.39	6.9e-05	3.34E-03
122	GO:0050776	regulation of immune response	312	132	99.91	6.9e-05	3.34E-03
123	GO:0006928	cellular component movement	824	314	263.86	7.0e-05	3.34E-03
124	GO:0050708	regulation of protein secretion	103	52	32.98	7.1e-05	3.34E-03
125	GO:0048514	blood vessel morphogenesis	304	129	97.35	7.1e-05	3.34E-03
126	GO:0032943	mononuclear cell proliferation	161	75	51.56	7.2e-05	3.34E-03
127	GO:0071396	cellular response to lipid	161	75	51.56	7.2e-05	3.34E-03
128	GO:0002286	T cell activation involved in immune res...	38	24	12.17	7.7e-05	3.55E-03
129	GO:0035914	skeletal muscle cell differentiation	36	23	11.53	8.3e-05	3.79E-03
130	GO:0051172	negative regulation of nitrogen compound...	731	281	234.08	8.4e-05	3.81E-03
131	GO:1902105	regulation of leukocyte differentiation	149	70	47.71	9.0e-05	4.05E-03
132	GO:0048660	regulation of smooth muscle cell prolife...	70	38	22.42	9.2e-05	4.11E-03
133	GO:0016310	phosphorylation	1217	448	389.71	9.4e-05	4.16E-03
134	GO:0090087	regulation of peptide transport	82	43	26.26	9.6e-05	4.16E-03
135	GO:0071346	cellular response to interferon-gamma	32	21	10.25	9.6e-05	4.16E-03
136	GO:0010647	positive regulation of cell communicatio...	715	275	228.96	9.6e-05	4.16E-03
137	GO:0048856	anatomical structure development	2457	863	786.79	0.00010	4.27E-03
138	GO:0045892	negative regulation of transcription, DN...	613	239	196.3	0.00010	4.27E-03
139	GO:0046651	lymphocyte proliferation	160	74	51.24	0.00011	4.54E-03

140	GO:0002292	T cell differentiation involved in immun...	26	18	8.33	0.00011	4.54E-03
141	GO:0002293	alpha-beta T cell differentiation involv...	26	18	8.33	0.00011	4.54E-03
142	GO:0001525	angiogenesis	247	107	79.1	0.00011	4.54E-03
143	GO:0045655	regulation of monocyte differentiation	8	8	2.56	0.00011	4.54E-03
144	GO:0030856	regulation of epithelial cell differenti...	52	30	16.65	0.00012	4.88E-03
145	GO:0046632	alpha-beta T cell differentiation	52	30	16.65	0.00012	4.88E-03
146	GO:0016525	negative regulation of angiogenesis	41	25	13.13	0.00013	5.08E-03
147	GO:0008202	steroid metabolic process	135	64	43.23	0.00013	5.08E-03
148	GO:1902679	negative regulation of RNA biosynthetic ...	629	244	201.42	0.00013	5.08E-03
149	GO:0051048	negative regulation of secretion	78	41	24.98	0.00013	5.08E-03
150	GO:0002764	immune response-regulating signaling pat...	153	71	48.99	0.00013	5.08E-03
